# Cellular Modifications of Rhodococci Exposed to Separate and Combined Effects of Pharmaceutical Pollutants

**DOI:** 10.3390/microorganisms10061101

**Published:** 2022-05-26

**Authors:** Irina Ivshina, Grigory Bazhutin, Semyon Tyan, Maxim Polygalov, Maria Subbotina, Elena Tyumina

**Affiliations:** 1 Perm Federal Research Center Ural Branch Russian Academy of Sciences, 13a Lenin Street, 614990 Perm, Russia; sniffedbybadger@gmail.com (G.B.); mapeyc@gmail.com (M.P.); ms.mari_999@mail.ru (M.S.); tyumina@psu.ru (E.T.); 2 Department of Microbiology and Immunology, Perm State National Research University, 15 Bukirev Street, 614990 Perm, Russia; vviolent00@mail.ru

**Keywords:** nonsteroidal anti-inflammatory drugs (NSAIDs), ibuprofen, meloxicam, naproxen, pharma pollutants, *Rhodococcus* spp., stress response, adaptation

## Abstract

Actinomycetes of the genus *Rhodococcus* (class Actinomycetia) are dominant dwellers of biotopes with anthropogenic load. They serve as a natural system of primary response to xenobiotics in open ecosystems, initiate defensive responses in the presence of pollutants, and are regarded as ideal agents capable of transforming and degrading pharmaceuticals. Here, the ability of selected *Rhodococcus* strains to co-metabolize nonsteroidal anti-inflammatory drugs (ibuprofen, meloxicam, and naproxen) and information on the protective mechanisms of rhodococci against toxic effects of pharmaceuticals, individually or in a mixture, have been demonstrated. For the first time, *R*. *ruber* IEGM 439 provided complete decomposition of 100 mg/L meloxicam after seven days. It was shown that versatile cellular modifications occurring at the early development stages of nonspecific reactions of *Rhodococcus* spp. in response to separate and combined effects of the tested pharmaceuticals included changes in electrokinetic characteristics and catalase activity; transition from unicellular to multicellular life forms accompanied by pronounced morphological abnormalities; changes in the average size of vegetative cells and surface area-to-volume ratio; and the formation of linked cell assemblages. The obtained data are considered as adaptation mechanisms in rhodococci, and consequently their increased resistance to separate and combined effects of ibuprofen, meloxicam, and naproxen.

## 1. Introduction

Along with the “great challenges” (e.g., climate change, water, food and energy security, depletion of natural resources, loss of biodiversity, and soil degradation) that humanity is facing in the current century, a new global challenge of pharmaceutical pollution poses a real and growing threat to human health and natural ecosystems [1,2,3,4,5].

The intensively developing pharmaceutics and novel drugs brought to the market, exponentially growing annual consumption of pharmaceuticals, enhanced access to medicines, and the lack of strict regulation of their use, together with the limited infrastructure of pharmaceutical waste management, lead inevitably to the ingress of active pharmaceutical ingredients into soil, sediments, wastewater, surface, ground, and even drinking water [6,7,8,9].

Recent global monitoring of pharmaceutically polluted rivers in 104 countries has revealed that the most frequently detected pharmaceutical drugs are analgesics (paracetamol), antiepileptics and antidepressants, antihistamines, antidiabetic agents (associated with a sedentary lifestyle of people due to urbanization), nonsteroidal anti-inflammatory drugs (diclofenac, ibuprofen, meloxicam, and naproxen), and antimicrobials [10]. The actual cumulative concentrations of the vast majority of them in surface waters often significantly exceeded the safe target levels. Given that organisms in natural ecosystems are simultaneously exposed to a cocktail of pharmaceutical ingredients, the environmental risks to human health, ecosystems, and plant and animal gene pools may well be much higher as a result of the toxicological interaction of these complex mixtures (for which the total concentration can amount to 297 μg/L [10]).

In this regard, one of the main socio-economic tasks is to develop scenarios, minimizing the negative impacts of pharmaceutical pollutants on natural biota and natural ecosystems’ sustainability. Studies in this area should focus on adequate measures to prevent and reduce environmental risks from pharmaceutical pollution [11]. To date, the weakest link is still the knowledge deficit of the negative impacts of these novel environmental micropollutants on microbiota, human, and animals. As microorganisms of polluted environments act as a primary response system to any xenobiotic loading in natural ecosystems, initiate the adaptive responses to pollution, and trigger the mechanisms of their detoxification and decomposition at earlier stages, the research efforts should primarily focus on an in-depth study of peculiar interactions of microorganisms with pharmaceutical pollutants [11,12,13]. This is essential for better understanding of protective mechanisms of native microbiota from real adverse effects of anthropogenic ecotoxicants and developing effective ways for their neutralization and removal from aquatic and terrestrial ecosystems.

In our previous studies, we observed biodegradation of pharmaceutical pollutants by bacteria of the genus *Rhodococcus* (class Actinomycetia)—a group of extremely tolerant mycolic acid-containing nocardioform actinomycetes, which are structurally, physiologically, and biochemically fit for decomposition of lipophilic organic compounds. Rhodococci are among the dominant microorganisms in anthropogenically disturbed biotopes and also participate in their restoration [14,15,16,17,18]. The ubiquity and localization of *Rhodococcus* spp. in extreme ecosystems, as well as their high biotechnology potential (including polluted site bioremediation), are due to their properties such as rigid hydrophobic cell walls and high affinity for hydrophobic substrates, a typically bacterial growth pattern, production of carotenoid pigments, oxidoreductase activity towards complex aromatic substrates, biosurfactant synthesis, low levels of endogenous respiration, tendency to cell adhesion and surface colonization, and oligotrophy for carbon, nitrogen, and sulfur sources [19,20,21].

Earlier, we have first shown the ability of individual members of the genus *Rhodococcus* for complete biodegradation of analgesic and spasmolytic agents, including paracetamol [22], drotaverine [23,24], and acetylsalicylic acid [25], as well as highly toxic nonsteroidal anti-inflammatory drugs (NSAIDs) diclofenac [26], ketoprofen [27], and ibuprofen [28].

The goal of this work was to study the ability of rhodococci to bioconvert individual NSAIDs (ibuprofen, meloxicam, and naproxen), most often detected in the environment, and to investigate the mechanisms of separate and combined impact of these NSAIDs on bacterial cells.

## 2. Materials and Methods

### 2.1. Chemicals

This study used ibuprofen, a propionic acid derivative ((RS)-2-[4-(2-methylpropyl)phenyl]propanoic acid, C_13_H_18_O_2_, CAS 15687-27-1); meloxicam, a derivative of oxicam ((4-hydroxy-2-methyl-N-(5-methyl-2-thiazolyl)-2H-1,2-benzothiazine-3-carboxamide-1,1-dioxide, C_14_H_13_N_3_O_4_S_2_, CAS 71125-38-7); and naproxen, a propionic acid derivative ((S)-6-methoxy-α-methyl-2-naphthaleneacetic acid in the form of sodium salt, C_14_H_14_O_3_, CAS 22204-53-1) (Sigma-Aldrich, St. Louis, MO, USA; BLD Pharmatech Ltd., Shanghai, China). Chemical reagents, including acetonitrile, methanol, chloroform, and ethanol were of chemical, analytical, or extra-pure grades (Cryochrome, Saint-Petersburg, Russia; Ekos-1, Moscow, Russia; Sigma-Aldrich, St. Louis, MO, USA). 

### 2.2. Strains

As effective biodegraders of ibuprofen, meloxicam, and naproxen, we used previously selected *Rhodococcus* strains with highly active oxygenases, namely *R*. *cerastii* IEGM 1278 [28,29], *R*. *ruber* IEGM 439, and *R*. *rhodochrous* IEGM 63 from the Regional Specialised Collection of Alkanotrophic Microorganisms (acronym IEGM, the large-scale research facilities (USU) # 73559, the core facilities (CKP) # 480868, the World Federation for Culture Collections (WFCC) # 285; http://www.iegmcol.ru/strains, accessed on 27 April 2022). The strains were selected considering their geography and source of isolation as well as for the well-known catalytic activities of rhodococci against complex organic compounds [30].

### 2.3. Culture Conditions

Bacteria were cultivated in mineral salt medium, as described earlier [25]. The initial concentrations (100 mg/L) of medicinal substances (ibuprofen, meloxicam, and naproxen) were chosen based on their actual environmental concentrations detected in water and soil. To study the combined action of NSAIDs, a mixture of ibuprofen, meloxicam, and naproxen (100 mg/L each) was used. Glucose (0.1 g/L) was used as a co-substrate. Rhodococci pre-grown in nutrient broth for three days and washed three times with 10 mM phosphate-buffered saline (pH 7.0) were used to inoculate the medium (OD_600_~0.4). The experiments were carried out in 250 mL Erlenmeyer flasks containing 100 mL of the mineral medium (160 rpm, 28 °C). Under these cultivation conditions, experiments on NSAID biodegradation were performed, and physicochemical, physiological, and morphometric responses of rhodococci exposed to NSAIDs were analyzed. The controls were (i) mineral salt medium plus individual NSAIDs (to assess the abiotic degradation of NSAIDs); (ii) mineral salt medium plus glucose and living bacterial cells without NSAIDs (to distinguish responses of rhodococci exposed to NSAIDs from those in the presence of glucose alone). 

### 2.4. Respirometry

Bacterial accumulation (μL) of CO_2_ released during NSAID biodegradation was assessed using a 10-channel Micro-Oxymax^®^ respirometer (Columbus Instruments, Columbus, OH, USA). The CO_2_ release was automatically recorded every 32 min for 7 days.

### 2.5. Microscopy

Cells were visualized using an Axio Imager M2 optical microscope (Carl Zeiss Microscopy GmbH, Jena, Germany) in phase-contrast and fluorescent modes. The images were captured using cameras such as Axoicam 506 Color and Zen Blue 3.1 (Carl Zeiss Microscopy GmbH, Jena, Germany). The morphometric parameters of bacterial cells were investigated using a unique combined scanning system consisting of an Olympus FV 1000 confocal laser scanning microscope (CLSM) (Olympus Corporation, Tokyo, Japan) and an Asylum MFP-3D atomic force microscope (AFM) (Asylum Research, Santa Barbara, CA, USA). Sample preparation and AFM scanning procedure were carried out according to Kuyukina et al. [31]. For that purpose, the cell suspension (15–20 µL) was stained with a two-component fluorescent dye LIVE/DEAD^®^ *Bac*Light^TM^ Bacterial Viability Kit (Invitrogen, Carlsbad, CA, USA). AFM scanning of the preparations was performed in a semi-contact mode in air at frequency of 0.2 Hz using an AC240TS silicon cantilever with resonance frequency of 50–90 kHz, spring constant of 0.5−4.4 N/m, and the curvature radius of the probe at 9 nm. The root-mean-square surface roughness and dimensions (length and width) of living cells were calculated using the Igor Pro 6.22A (WaveMetrics, Portland, OR, USA) software. Cell volume and surface area were calculated using the formulas in [32]. 

### 2.6. Zeta Potential

The surface charge of bacterial cells was estimated by measuring their electrokinetic potentials (zeta potentials) by the dynamic light scattering technique using a ZetaSizer Nano ZS analyzer (Malvern Instruments, Malvern, UK) and the Malvern ZetaSizer software, v. 2.2 (Malvern Instruments, Malvern, UK). Cells grown in the mineral salt medium in the presence of (i) individual NSAIDs and glucose, (ii) a mixture of NSAIDs and glucose, or (iii) only glucose were washed twice with phosphate buffer (pH 7.0) and resuspended in 0.1 M KNO_3_ (pH 7.0) until OD_600_ 0.2 was reached. Measurements were performed in a U-shaped cuvette in phosphate buffer (pH 7.4) at 25 °C. 

### 2.7. Catalase Activity

The catalase activity of bacterial cells exposed to ecotoxicants was evaluated spectrophotometrically [33]. Briefly, bacterial cells grown in the presence of individual NSAIDs or their mixture and glucose were centrifuged at 3000 rpm for 5 min, washed with phosphate buffer (pH 7.0), and resuspended in the same buffer to OD_492_ of 0.2. A 0.00125 M H_2_O_2_ solution (1 mL) was added to 200 µL of the obtained cell suspension and incubated for 10 min at room temperature. Then, 100 μL of 2N HCl solution was added to stop the process of hydrogen peroxide decomposition by catalase. A 0.025 M KI solution (1 mL) was added to the resulting mixture, gently mixed, and centrifuged at 3000 rpm for 15 min. In control samples, distilled water was used instead of cell suspensions. The absorbance of the supernatant was measured using a Lambda EZ201 spectrophotometer (Perkin-Elmer, Waltham, MA, USA) at 492 nm.

### 2.8. Analytical Methods

NSAIDs were detected using a Prominence LC-20A high-performance liquid chromatograph (Shimadzu, Tokyo, Japan) equipped with an SPD-M20A diode array detector and a Discovery^®^ C18 HPLC column, 5 μm particle size, 25 cm × 4.6 mm (Sigma-Aldrich, Supelco, St. Louis, MO, USA) [16]. Optimal conditions for NSAID detection were as follows: a mobile phase of phosphate buffer solution (pH 5.0 for naproxen and pH 3.5 for ibuprofen and meloxicam) and acetonitrile (40:60, *v*/*v*); flow rate, 0.5 mL/min; column temperature, 40 °C for naproxen and ibuprofen and 30 °C for meloxicam; injected volume, 20 µL; and detection wavelength, 254 nm. 

The experiments were performed in 3 to 30 replicates.

## 3. Results and Discussion

### 3.1. Biodegradation of Individual NSAIDs and Induced Morphological Changes of Biodegrading Strains

According to our data, rhodococci can degrade NSAIDs with different efficiencies (Table 1). On the seventh day of the experiment, a complete removal was observed only for meloxicam. Previously, we have shown the ability of rhodococci to completely biodegrade acetylsalicylic acid as the sole carbon and energy source and to co-metabolize diclofenac (with glucose), ketoprofen and ibuprofen (with *n*-hexadecane) [25,26,27,28]. Here, we confirmed for the first time the biodegradability of naproxen and meloxicam using *Rhodococcus* spp.

**Figure 1:**
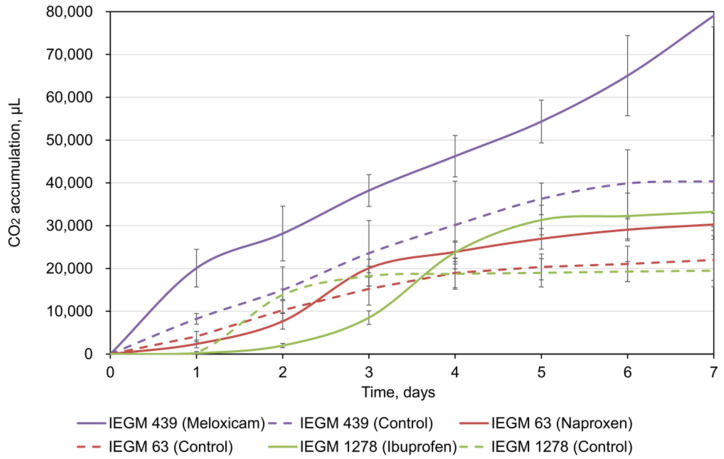
CO_2_ accumulation by rhodococci during NSAID bioconversion. The data are presented as mean values ± standard deviations (*n* = 3).

Respirometry analysis is one of the indicators of bacterial activity during biodegradation of complex organic substrates [26,28,34]. Our respirometry test data showed that the catalytic activity of rhodococci increased significantly (*p* < 0.05) in the presence of NSAIDs: the average values of CO_2_ accumulation by cells exposed to pharmaceuticals were 1.4–1.9 times higher than those in the controls (Figure 1). Interestingly, in the presence of meloxicam, no lag phase was observed for *R*. *ruber* IEGM 439 cells, while in the presence of naproxen and ibuprofen, the lag phases for all *Rhodococcus* strains tested were two and three days, respectively. A gradual decrease in CO_2_ release in the presence of naproxen (on the third day) and ibuprofen (on the fifth day) may suggest the formation and accumulation of toxic metabolites during biodegradation [28]. In addition, the maximum CO_2_ production was registered for meloxicam, exhibiting the highest biodegradability according to our data. 

Microscopic analyses demonstrated that NSAID exposure caused morphological changes in cells, namely in their length and width, as well as a decrease in surface-area-to-volume ratios (SA/V) (Table 2).

SA/V is an important parameter, indicating the bacterial ability to adapt to adverse habitats, including the pollutant load [35]. In our case, rhodococci sought to lower this parameter in order to reduce the contact area of cells with toxic NSAIDs. Evidently, this may also be explained by the influence of NSAIDs, acting as antibiotic agents and disturbing the biosynthesis of cell wall components [36,37].

### 3.2. Effects of Individual NSAIDs and Their Mixture on Rhodococcus cerastii IEGM 1278 Cells

In the environment, mixtures of various pharmaceutical compounds, including NSAIDs, have been detected [1,3,9]. In this regard, it is essential to take into account the effects of pharmaceutical cocktails on microorganisms. Below are the results of separate and combined effects of NSAIDs, as exemplified by *R*. *cerastii* IEGM 1278.

**Figure 2:**
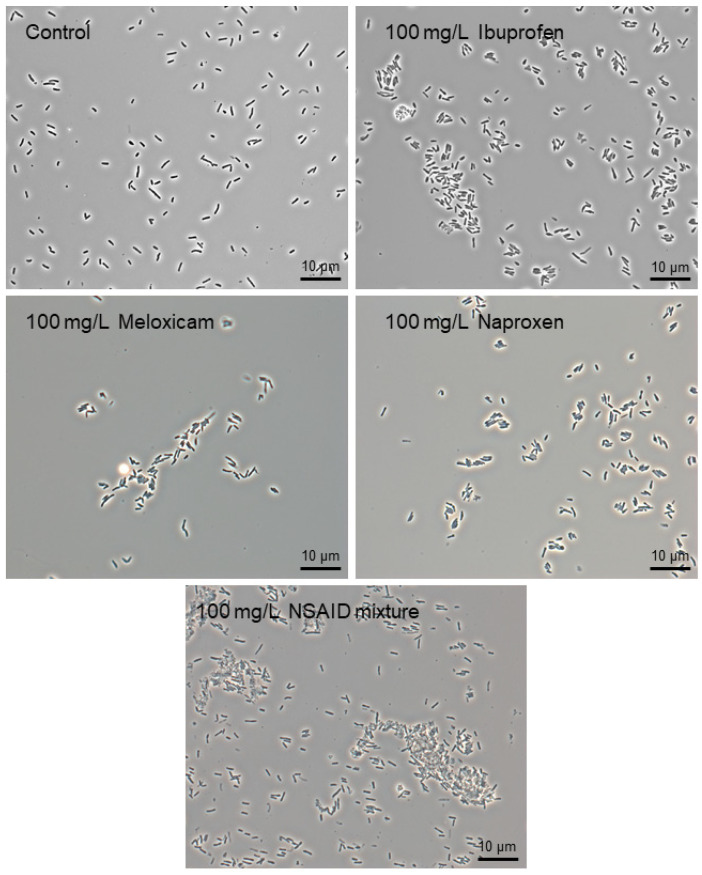
Phase-contrast images of *R*. *cerastii* IEGM 1278 grown in the presence of 0.1 g/L glucose (control), 0.1 g/L glucose and selected NSAIDs (ibuprofen, meloxicam, and naproxen), and their mixture. Cells were cultured for three days. × 1000.

In the presence of individual NSAIDs or their mixture, rhodococci are prone to cohesion (autoaggregation), forming aggregates of different size and shape (Figure 2). This mechanism is a universal adaptive response of rhodococci to both pharmaceutical pollutants [26,27,28] and other ecotoxicants present in the environment [38]. When aggregated, cells within these multicellular structures are structurally and physiologically distinct from planktonic cells. For example, aggregated cells provide coordinated behavior through cell-cell signaling, better access to growth resources, more progeny per initial, and enhanced production of exopolymeric substances [39,40]. It allows for the cells’ protection from toxic effects and their joint attack on a substrate for more effective degradation/transformation [41,42,43].

Auto-aggregation can be based on various physicochemical and molecular mechanisms [40,44]. An informative biophysical parameter of bacterial cells is their surface charge, dependent on biochemical composition of the cell surface (and a physiological state of cells) and evaluated by their electrokinetic potential (zeta potential). Zeta potential measurements of cells are useful for analyzing the state of the bacterial membrane and cell wall, whilst we used this value to assess the effects of NSAIDs on rhodococci.

**Figure 3:**
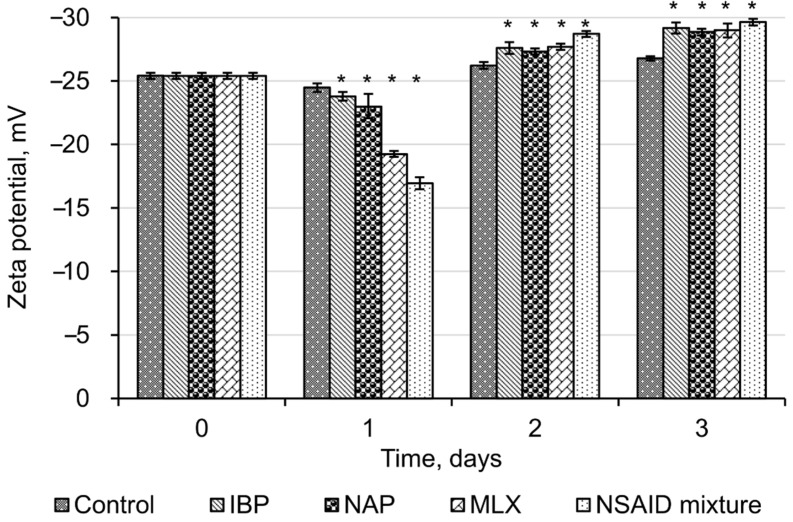
Zeta potential values of *R*. *cerastii* IEGM 1278. Cells were grown in the presence of 0.1 g/L glucose (control), 0.1 g/L glucose and selected NSAIDs (ibuprofen, IBP; naproxen, NAP; and meloxicam, MLX), and their mixture. The data are presented as mean values ± standard deviations (*n* = 10). Mean values are significantly different from the control: * *p* < 0.05.

The initial values of zeta potential for *R*. *cerastii* IEGM 1278 were −25.4 ± 0.25 mV (Figure 3). Negative zeta potential values of cell membranes at physiological pH values are probably due to the presence of ionogenic groups in phospholipids, proteins, and their conjugates with polysaccharides. In addition, negative zeta potential of *Rhodococcus* cells may also result from total mycolic acids present in the cell wall [45].

The initial exposure (one day) of all tested NSAIDs and their mixture reliably (*p* < 0.05) led to a decrease in zeta potential values of *R*. *cerastii* IEGM 1278. At the same time, the most significant changes in their surface charge were observed with meloxicam and a mixture of NSAIDs, with zeta potential values decreasing from the initial −25.4 mV to −19.2 mV and −16.9 mV, respectively. Neutralization of the negative charge of the cell wall of NSAID-exposed rhodococci indicates a significant release of potassium ions, which are mainly localized intracellularly. In addition, we have previously shown that the bactericidal effect of NSAIDs is accompanied by a change in the membrane permeability, so that temporary pores in the plasma membrane are formed and ions are released—less negative zeta potential values correlate with greater membrane permeability [28]. It is known that ion channels play an important role in bacterial adaptation to endogenous and exogenous stress [46]. Of particular interest are potassium ion channels which regulate intercellular interactions, stress resistance, and biofilm formation in Gram-positive *Bacillus subtilis* [47,48,49]. Thus, taking into account the function of potassium ion channels, we may consider the decrease in zeta potential on the first day of the experiment as a prerequisite of bacterial aggregation (see Figure 2).

**Figure 4:**
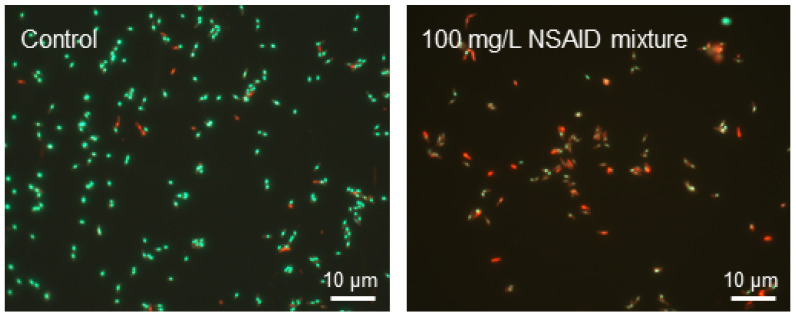
Fluorescent microscopy of *R*. *cerastii* IEGM 1278 cells grown for three days in the presence of 0.1 g/L glucose (control), or 0.1 g/L glucose and a mixture of NSAIDs. Cells were stained with LIVE/DEAD Bacterial Viability kit (live/dead = green/red, respectively).

On the second and the third day of cell incubation with NSAIDs, the average zeta potential values shifted to negative values (up to −29.6 ± 0.26 mV in the presence of NSAID mixture). Apparently, this is due to redistribution of phosphatidylserine, carrying a negatively charged carboxyl group, from the inner lipid layer of the plasmalemma to the outer one [50]. The occurrence of phosphatidylserine in the outer lipid monolayer of the cell membrane is one of the early markers of apoptosis and decreased cell viability, as confirmed by fluorescence microscopy data (Figure 4). We assume that NSAIDs are likely to bind to the membrane, causing a change in membrane permeability due to the displacement of Ca^2+^ and Mg^2+^ and this leads to membrane disturbance [51]. In addition, dead or damaged cells have an enhanced leakage of charged molecules into the surroundings, increasing the zeta potential [52]. 

Thus, the initial contact of NSAIDs with bacterial cells results from the electrostatic interaction of oppositely charged molecules; and there are no significant differences in the nature of such interaction for different NSAIDs.

**Figure 5:**
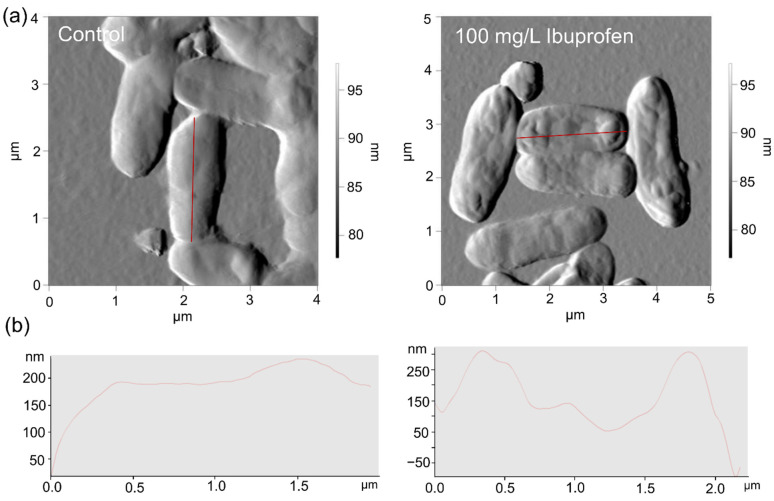
AFM (**a**) images and profiles (**b**) of *R*. *cerastii* IEGM 1278. Cells were grown for three days with 0.1 g/L glucose (control), or 0.1 g/L glucose and 100 mg/L ibuprofen.

AFM/CLSM scanning results showed the changed nano-geometric characteristics of the cell surface of rhodococci exposed to NSAIDs. Thus, in the presence of ibuprofen, the increased mean square roughness of cells was observed. In the control, the roughness values corresponded to 218 ± 32.5 nm, while under the influence of ibuprofen, this value increased to 289 ± 29.4 nm (Figure 5).

**Figure 6:**
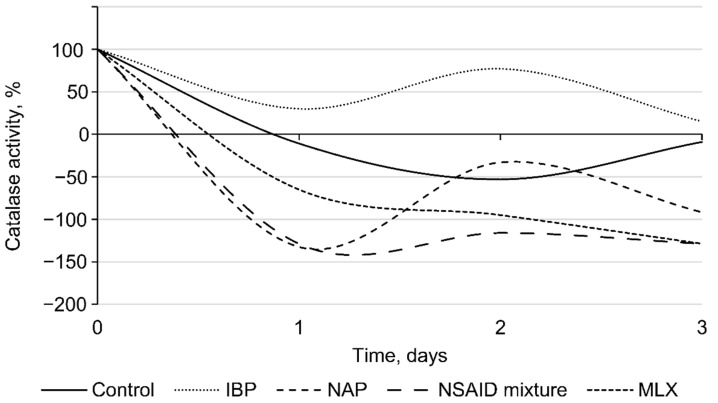
Catalase activity of *R*. *cerastii* IEGM 1278. Cells were grown in the presence of 0.1 g/L glucose (control), 0.1 g/L glucose and selected NSAIDs (ibuprofen, IBP; naproxen, NAP; and meloxicam, MLX), and their mixture. The data are presented as mean values ± standard deviations (*n* = 3).

In addition, auto-aggregation is an initial prerequisite to the formation of biofilms, protecting cells against various adverse factors, including oxidative stress [53]. According to our data, NSAIDs present in the environment induced an oxidative stress in bacterial cells, as evidenced by the levels of catalase activity of rhodococci (Figure 6). The initial catalase activity of *R*. *cerastii* IEGM 1278 was 0.66 relative units corresponding to 100%. On the first day of the experiment, a decrease in catalase activity was observed for all samples. At the same time, the most significant changes (*p* < 0.05) were recorded in the presence of naproxen and a mixture of NSAIDs. On the second day, the catalase activity of NSAID-exposed rhodococci increased and then slightly (smoothly) declined by day three. The toxic effects of NSAIDs apparently induce the alternate involvement of enzymatic (catalase) cell protection and an alternative non-enzymatic system (polysaccharides, trehalose mycolates), allowing for optimal allocation of resources and nutrients, while making it possible not to spend them consistently on the energy-consuming process of synthesis of antioxidant enzymes. To put it another way, when the cell wall is intact, it protects bacteria from oxidative stress adequately, but when the cell wall is destabilized, enzymes, particularly catalase, are responsible for efficient defense against oxidative stress [33]. In our case, on the second day, an increase in catalase activity was observed, which is probably associated with induced cell permeability, cell damage, and an increase in zeta potential (see Figure 3 and Figure 4).

## 4. Conclusions

Pharmaceutical pollution is becoming a major worldwide concern and has severe impact on human health and biota. To address this problem, it is important to know how pharmaceuticals behave in the environment and interact with natural microorganisms, acting as a primary response system under xenobiotic load. *Rhodococcus* spp. are typical inhabitants of polluted areas, with a wide variety of adaptive capacities and abilities to decompose numerous complex organic contaminants. In the present study, the adaptive reactions of *Rhodococcus* strains to toxic effects of individual NSAIDs (ibuprofen, meloxicam, and naproxen) and their mixture were revealed. These responses have a multifaceted complex nature and are represented not only in cytology of *Rhodococcus*. They manifest in universal morphological and physiological abnormalities of cells as well as at other levels of cellular organization, for example, in a variety of enzymes, including an antioxidant defense system of bacterial cells.

## Figures and Tables

**Table 1:** Biodegradation of NSAIDs by rhodococci on the seventh day of the experiment.

Strain	NSAID	Biodegradation, %
*R*. *cerastii* IEGM 1278	100 mg/L ibuprofen	7.8 ± 3.97
*R*. *ruber* IEGM 439	100 mg/L meloxicam	99.3 ± 1.37
*R*. *rhodochrous* IEGM 63	100 mg/L naproxen	46.6 ± 3.45

The data are presented as mean values ± standard deviations (*n* = 3).

**Table 2:** Morphometric characteristics of rhodococci exposed to NSAIDs.

Strain	Variant	Length, μm	Width, μm	Volume (V), μm^3^	Surface Area (SA), μm^2^	SA/V, μm^−1^
Ibuprofen						
*R*. *cerastii* IEGM 1278	Control	3.7 ± 0.25	1.0 ± 0.07	2.9 ± 0.41	12.5 ± 1.19	4.6 ± 0.28
	100 mg/L	2.8 ± 0.29 **	1.1 ± 0.10 *	2.6 ± 0.42	11.5 ± 1.12 *	4.4 ± 0.28
Meloxicam						
*R*. *ruber* IEGM 439	Control	3.3 ± 0.28	0.9 ± 0.10	1.9 ± 0.46	10.1 ± 0.46	5.3 ± 0.54
	100 mg/L	2.7 ± 0.18 *	1.0 ± 0.10 *	2.0 ± 0.45	9.8 ± 1.30	4.9 ± 0.39
Naproxen						
*R*. *rhodochrous* IEGM 63	Control	1.9 ± 0.10	1.0 ± 0.07	1.4 ± 0.21	7.12 ± 0.68	5.3 ± 0.28
	100 mg/L	1.6 ± 0.17 *	1.1 ± 0.06 **	1.6 ± 0.29 *	7.7 ± 0.91	4.8 ± 0.31 *

Cells were cultured for three days. The data are presented as mean values ± standard deviations (*n* = 30). Mean values are significantly different from the control: * *p* < 0.05, ** *p* < 0.01.

## Data Availability

The data generated during the current study are available from the corresponding author on reasonable request.
